# Lineage sorting in multihost parasites: *Eidmanniella albescens* and *Fregatiella aurifasciata* on seabirds from the Galapagos Islands

**DOI:** 10.1002/ece3.1587

**Published:** 2015-07-16

**Authors:** Jose L Rivera-Parra, Iris I Levin, Kevin P Johnson, Patricia G Parker

**Affiliations:** 1 Department of Biology and Whitney R. Harris World Ecology Center, University of Missouri – St LouisSt Louis, Missouri, 63110; 2 Facultad de Geología y Petróleos, Departamento de Petróleos, Escuela Politécnica Nacional del EcuadorQuito, Ecuador; 3 Illinois Natural History Survey, University of IllinoisChampaign, Illinois, 61820; 4 Saint Louis Zoo WildCare Institute, One Government DriveSaint Louis, Missouri, 63110

**Keywords:** Chewing lice, cryptic speciation, lineage sorting, parasites, seabirds

## Abstract

Parasites comprise a significant percentage of the biodiversity of the planet and are useful systems to test evolutionary and ecological hypotheses. In this study, we analyze the effect of host species identity and the immediate local species assemblage within mixed species colonies of nesting seabirds on patterns of genetic clustering within two species of multihost ectoparasitic lice. We use three genetic markers (one mitochondrial, *COI,* and two nuclear, *EF1-α* and *wingless*) and maximum likelihood phylogenetic trees to test whether (1) parasites show lineage sorting based on their host species; and (2) switching of lineages to the alternate host species depends on the immediate local species assemblage of individual hosts within a colony. Specifically, we examine the genetic structure of two louse species: *Eidmanniella albescens,* infecting both Nazca (*Sula granti*) and blue-footed boobies (*Sula nebouxii*), and *Fregatiella aurifasciata*, infecting both great (*Fregata minor*) and magnificent frigatebirds (*Fregata magnificens*). We found that host species identity was the only factor explaining the patterns of genetic structure in both parasites. In both cases, there is evident genetic differentiation depending on the host species. Thus, a revision of the taxonomy of these louse species is needed. One possible explanation of this pattern is extremely low louse migration rates between host species, perhaps influenced by fine-scale spatial separation of host species within mixed colonies, and low parasite infrapopulation numbers.

## Introduction

Parasites comprise a significant percentage of the planet's biodiversity (Koh et al. 2004; Whiteman and Parker 2005). There is variation in the nature of these relationships, with an extreme of complete dependence of the parasite on the host, such as malarial protozoan parasites and ectoparasitic lice and mites (Price et al. 2003; Valkiûnas 2005). This study reports our studies of ectoparasitic chewing lice, which are obligate parasites that depend on the resources and microclimate of the host to survive (Price et al. 2003). Parasites with a life history strongly tied to the host have proved to be excellent systems in which to pose questions on the generation and maintenance of diversity and on mechanisms of speciation (Johnson et al. 2003a,b; Whiteman and Parker 2005; Whiteman et al. 2007; Hughes et al. 2007). Moreover, because their populations are fragmented into small infrapopulations, with varying degrees of connectivity depending on both host and parasite dispersal capabilities, permanent parasites can be good models in which to examine island biogeography and meta-community dynamics (Weckstein 2004; Banks et al. 2006; Whiteman and Parker 2005; Whiteman et al. 2007).

Johnson et al. (2003a,b) and Huyse et al. (2005) summarized the modes of parasite speciation as follows: (1) cospeciation, where speciation in parasites follows speciation in the hosts; (2) host switching, where a parasite colonizes a novel host and limited gene flow leads to later speciation; and (3) parasite duplication, where structure in the host population limits gene flow in the parasites. Among these, the most studied mechanism is cospeciation. Studies such as Hughes et al. (2007) and Kaewmongkol et al. (2011) have provided examples of parasites matching the evolutionary history of their hosts. Thus, restriction of host gene flow can similarly limit parasite gene flow, resulting in parasite speciation. Studies analyzing such coevolutionary patterns have inferred host switching when incongruent phylogenetic trees of hosts and parasites are observed (e.g., Hughes et al. 2007). Studies focusing on parasite duplication, or parasite differentiation, even when the host has not differentiated to the point of separate species designation, are rare. Whiteman et al. (2007) found that in the Galapagos hawk, which has a significantly structured population across the archipelago, ectoparasitic lice show higher genetic differentiation and genetic isolation, which may be early steps of geographic differentiation and later speciation. While structure among hawk island populations is best predicted by distance between islands, structure among their louse populations is best predicted by structure among hawk populations (Koop et al. 2014). The situation becomes more complex in cases where a parasite species infects more than one host species, and few studies of parasites have examined parasite divergence in these cases.

Even in groups of parasites where most species infect only one host species (e.g., Johnson et al. 2002; Barrett et al. 2008), there are examples of parasites infecting multiple host species (e.g., avian malaria in African forest birds, Njabo et al. 2011; dove feather lice, Johnson et al. 2002). One possible scenario is that these are cases of cryptic species where parasites are morphologically identical and there are host-specific lineages (Poulin and Keeney 2008; Malenke et al. 2009). Cryptic species of parasites might be relatively common, and estimates of host specificity might change if genetic studies of multihost parasite species were performed. McCoy et al. (2003, 2005) analyzed a common and shared tick species, which infects seabirds, and found clear evidence of lineage sorting (or race formation) based on the host that they were infecting. The genetic differentiation depended on the extent and type of interactions among individuals within and between host species (McCoy et al. 2005). Thus, overlapping host species with a relatively high degree of interaction (e.g., nesting next to each other in a mixed colony) have the potential to limit the genetic differentiation of the parasites. In this study, we analyze how host interactions can shape parasite diversity, focusing on obligate ectoparasitic lice that depend entirely on their hosts for survival and transmission (Clayton et al. 1992; Price et al. 2003; Huyse et al. 2005; Nieberding and Olivieri 2007).

**Figure 1:**
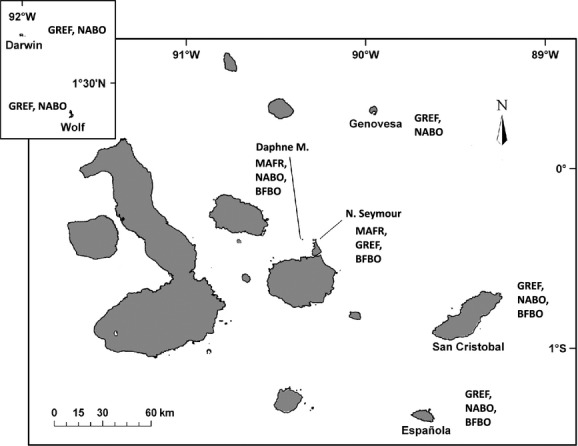
Map of the Galapagos archipelago showing the sampled islands and the local host community composition. Sampled Islands include Darwin, Wolf, Genovesa, North Seymour, Daphne Major, Española, and San Cristobal. Species codes are as follows: GREF (*Fregata minor*); MAFR (*Fregata magnificens*); NABO (*Sula granti*); and BFBO (*Sula nebouxii*). The number of parasites sequenced from each island for each host is shown in the parentheses.

We studied the genetic structure of two multihost ectoparasitic lice: *Eidmanniella albescens,* parasitic on boobies (*Sula* spp.), and *Fregatiella aurifasciata,* parasitic on frigatebirds (*Fregata* spp.). Neither louse species shows any morphological differentiation between populations on different host species (José Luis Rivera-Parra, pers. obs.). Populations of these parasites were studied in host populations that occur in the Galapagos archipelago (Fig. 1). Both parasites, *Eidmanniella albescens* and *Fregatiella aurifasciata*, are obligate ectoparasitic lice (Phthiraptera) from the suborder Amblycera, members of which have relatively high dispersal capabilities, feed from tissue and blood of the host, and are transmitted via direct contact among hosts (Price et al. 2003). Both parasites are relatively uncommon, with a prevalence of 35% for *F. aurifasciata* and 27% for *E. albescens,* and a median intensity of infection of 1.8 individuals per infected host for both parasites (Rivera-Parra et al. 2014, studying the same host species). *Fregatiella aurifasciata* is found on both species of frigatebirds in the archipelago (Palma and Peck 2013), the magnificent frigatebird (*Fregata magnificens*) and the great frigatebird (*Fregata minor*). *Eidmanniella albescens* is found on two of the three species of boobies in the archipelago (Palma and Peck 2013; Rivera-Parra et al., 2014), the blue-footed booby (*Sula nebouxii*) and the Nazca booby (*Sula granti*). However, *E. albescens* is not found on the red-footed booby in the Galapagos (*Sula sula*), even though this louse species is reported from red-footed boobies elsewhere (Price et al. 2003). The absence of this louse on red-footed boobies in the Galapagos is also surprising, given that the red-footed booby is sympatric with the other hosts on several islands.

Regarding host population genetic structure (which is a proxy for host intraspecies interisland connectivity), Levin and Parker (2012) found no genetic structure in the great frigatebird among five island populations within the archipelago, similar to the findings of Taylor et al. (2011) on three colonies of blue-footed boobies. On the other hand, in five island populations of Nazca boobies, there is evidence of genetic structure between several pairs of islands, resulting in three distinct genetic clusters (Levin and Parker 2012). To the best of our knowledge, there are no studies describing the intra-archipelago genetic structure of the magnificent frigatebird. However, Hailer et al. (2011) found that the Galapagos population as a whole is isolated from its conspecifics. All the host species overlap in parts of their range and have different degrees of spatial overlap in mixed nesting colonies. Satellite tracking studies complement population genetic approaches and also reveal connectivity of seabird populations across large geographic scales (e.g., Weimerskirch et al. 2006).

The goals of our research were to test whether (1) multihost parasites in a potentially highly connected system are the same species or there is evidence of genetic lineage sorting based on the host species; and (2) the degree of spatial overlap of potential hosts explains patterns of genetic clustering. Our specific predictions were that (1) there will be evidence of lineage sorting depending on the host species; and (2) such evidence will be weaker on islands where the hosts overlap spatially in mixed colonies.

## Materials and Methods

### Ectoparasite collection – dust ruffling

Adult (*n* = 67) and juvenile (*n* = 9) birds were captured by hand when they were sleeping or incubating, in which case, the chicks or eggs were covered from the sun. In order to avoid double-sampling, we put temporary marks on their beaks (details in Rivera-Parra et al. 2014). We followed a modified dust ruffling protocol (based on Walther and Clayton 1997). We applied a standardized amount (∼6 g) of powder to each host throughout the body, ruffling a maximum of three times, and waited a standard time (2 min) between bouts of ruffling. We stored the collected ectoparasites in 95% ethanol. Louse identification followed the key and information of host–parasite association found in Price et al. (2003) and Palma and Peck (2013).

From each sampled host, we described the immediate host community assemblage within a colony by recording the identity of and distance to the nearest neighbor and the species composition of nests within 10 m. This measure was used as an estimate of interspecies interaction and a measure of breeding density. Figure1 summarizes the islands sampled and the local host species composition relevant to this study.

### Molecular analysis

We extracted DNA from individual lice using the voucher method (Cruickshank et al. 2001) using a Macherey-Nagel Tissue extraction kit (Macherey-Nagel, CO., Düren, Germany). We followed the kit protocol, with the following modifications: We used 20 *μ*L of proteinase K and incubated the whole body for 72 h after making a partial cut between the head and the thorax, keeping the head attached to the body (J. Weckstein, pers. comm.) and performed two sequential DNA elutions, each with 20 *μ*L of warm buffer. We chose one mitochondrial marker, cytochrome oxidase subunit I (COI), which is a commonly used marker that is consistently informative in insects (Johnson et al. 2003a,b). We also analyzed two nuclear markers, the subunit alpha of the eukaryotic elongation factor (EF1-*α*) and *wingless,* which is associated with wing development in insects; both genes have been used in comparable species-level studies (Johnson et al. 2003a,b; Lin and Danforth 2004). We amplified the three gene regions using 1 *μ*L of total genomic DNA in a 25 *μ*L PCR with TaKaRa *Ex Taq* polymerase and reagents (TaKaRa Bio Inc., Shiga, Japan). The specific conditions were as follows: 1X MgCl_2_-free buffer (2.5 *μ*L; TaKaRa), 1.5 mmol/L of MgCl_2_ (1.5 *μ*L; TaKaRa), 0.2 mmol/L of each dNTP (2 *μ*L; TaKaRa), 0.08 mg/mL of BSA (0.2; Promega, Madison, WI, USA), and 0.625 units of TaKaRa *Ex Taq* DNA polymerase (0.125 *μ*L; TaKaRa). We amplified *COI* using the primers L6625 (5'-COG GAT CCT TYT GRT TYT TYG GNC AYC C-3') and H7005 (5' –CCG GAT CCA CAN CRT ART ANG TRT CRT G-3'; Hafner et al. 1994). The specific amplification conditions were initial denaturation at 94°C for 2 min, then 35 cycles of 94°C for 30 sec, 46°C for 30 sec, and 72°C for 30 sec, and then a final extension at 72°C for 7 min. For EF1-*α*, we used the primers EF1-For3 (5'-GGN GAC AAY GTT GGY TTC AAC G-3') and Cho 10 (5'-AC RGC VAC KGT YTG HCK CAT GTC-3'; Danforth and Ji 1998). The specific PCR conditions were an initial denaturation for 4 min at 94°C, then 35 cycles of 94°C for 20 sec, 45°C for 30 sec, and 72°C for 50 sec, and then a final extension for 5 min at 72°C. In the case of *wingless,* we used the primers Lep wg1a (5'-GAR TGY AAR TGY CAY GGY ATG TCT GG-3') and Lep wg2a (5'-ACT ICG CAR CAC CAR TGG AAT GTR CA-3'; Hughes et al. 2007; Danforth et al. 2004), with reaction conditions of initial denaturation for 4 min at 94°C, then 35 cycles of 94°C for 45 sec, 50°C for 45 sec, and 72°C for 45 sec, and then a final extension for 5 min at 72°C. (GenBank accession numbers: COI KT238383-KT238469, KT238596-KT238710; EF1-*α* KT238470-KT238555, KT238711-KT238825; wingless KT238556-KT238595).

### Phylogenetic analyses

We used MEGA v5.0 (Tamura et al. 2011) to build maximum likelihood trees for each gene and to test for the best fitting model. We constructed maximum likelihood trees using a T92+I evolutionary model when analyzing *COI,* Jukes–Cantor for *EF1-α,* and a T92+G model for *wingless*, with 1000 bootstrap replicates. In order to root the *Eidmanniella albescens* trees for *COI* and *EF1-α,* we used a sequence from *Fregatiella aurifasciata* from the same genes. We did the same for the *F. aurifasciata* trees, using *E. albescens* sequences to root them. *F. aurifasciata* and *E. albescens* are considered closely related species that were once part of the same genus (Ryan and Price 1969). In the case of the *E. albescens* tree for *wingless,* we used reference sequences from GenBank of species from the same family (Menoponidae), specifically from *Heteromenopon psittacum* (GU569387.1; Yoshizawa and Johnson 2010) and *Trinoton querquedulae* (GU569385.1; Yoshizawa and Johnson 2010).

## Results

### Spatial distribution of hosts

In the case of frigatebirds, the only island included in this study where both species breed in sympatry is North Seymour (*n* = 30), where the sampled great frigatebirds nest in colonies that have an average of 1.8 nests within ten meters of the sampled nest, of which 0.55 nests correspond to magnificent frigatebirds and 1.25 nests correspond to conspecifics (great frigatebirds). On the other hand, the magnificent frigatebirds on North Seymour (*n* = 20) have an average of 3.95 nests within ten meters, of which one is a nest of great frigatebirds and 2.95 are magnificent frigatebirds.

The two booby species are sympatric on Española, San Cristobal, and Daphne Islands (Fig. 1). On Daphne and San Cristobal, the nests of blue-footed and Nazca boobies are not closely associated (no Nazca boobies nest within ten meters of a sampled blue-footed booby nest and vice versa). On Española, the Nazca boobies (*n* = 39) had an average density of 4.19 nests within ten meters of the focal nest, but none of these nests are of blue-footed boobies. The blue-footed boobies (*n* = 15) have an average of 6.82 nests within ten meters, of which 0.67 belong to Nazca boobies and 6.15 correspond to other blue-footed boobies (Rivera-Parra et al. 2014).

### Eidmanniella albescens

#### COI

**Figure 2:**
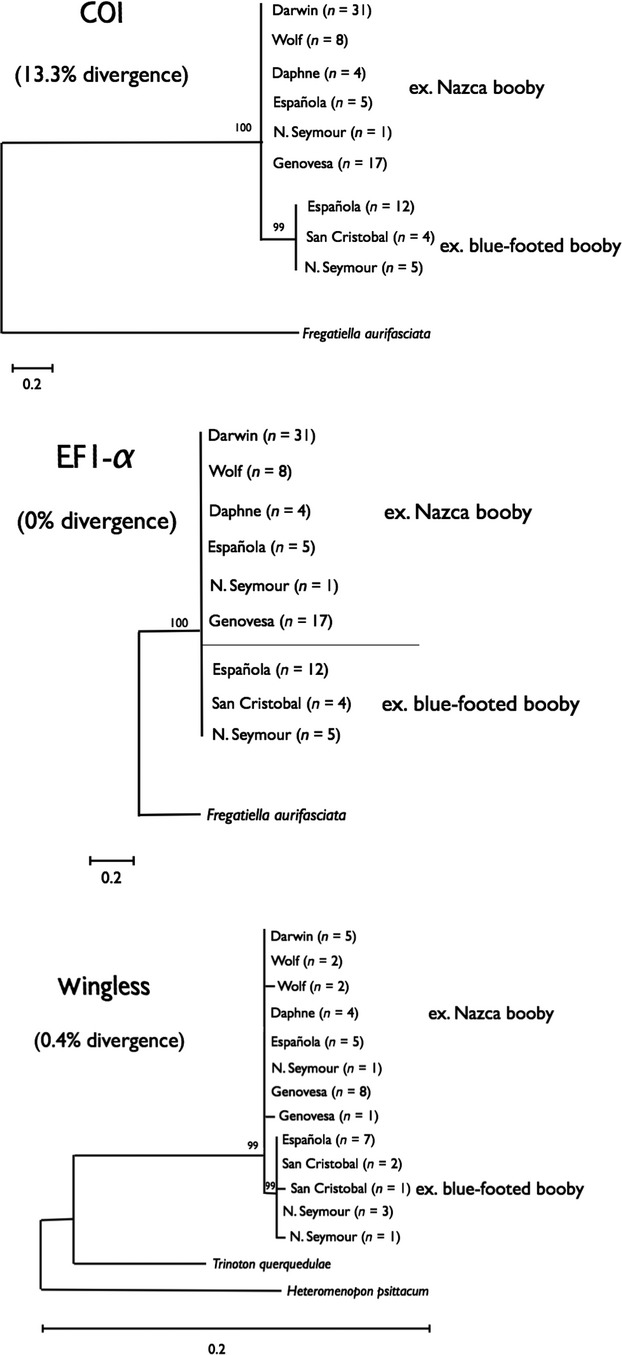
Maximum likelihood phylogenetic trees for the tree genetic markers, *COI, EF1-α,* and *wingless,* for *Eidmanniella albescens*. The number of parasites analyzed from each population is noted in parenthesis next to the island name.

We sequenced 87 individuals of *Eidmanniella albescens* and found complete lineage sorting by host species, thus revealing a Nazca booby lineage and a blue-footed booby lineage within these lice (Fig. 2). The genetic distance between these lineages is 13.0% or 39 bp in the sequenced 300-bp fragment. There was no genetic variation within either haplotype cluster at this region of COI.

#### EF1-*α*

There was no genetic variability in *EF1-α* across 270 bp of sequence among all the individuals of *E. albescens* (Fig. 2). Thus, it was not possible to detect any lineage sorting by host species at this locus.

#### Wingless

To further test the results found in *EF1-α,* we sequenced 348-bp *wingless* fragment in a subsample of 42 individuals of *E. albescens* (14 found on blue-footed boobies and 28 found on Nazca boobies, which represent a random sample across islands and are proportional to the sampled parasites). Unlike *EF1-α,* we did find evidence of lineage sorting using this nuclear marker (Fig. 2). Sequences of lice on different host species differed by 0.4% genetic distance, that is, by a single base across 348 bp. A transition in the position 77 of the amplified fragment sorted lice from Nazca booby versus blue-footed booby. The mean within-lineage genetic variability found in the Nazca booby lineage and the blue-footed booby lineage was 0.1%.

### Fregatiella aurifasciata

#### Coi

**Figure 3:**
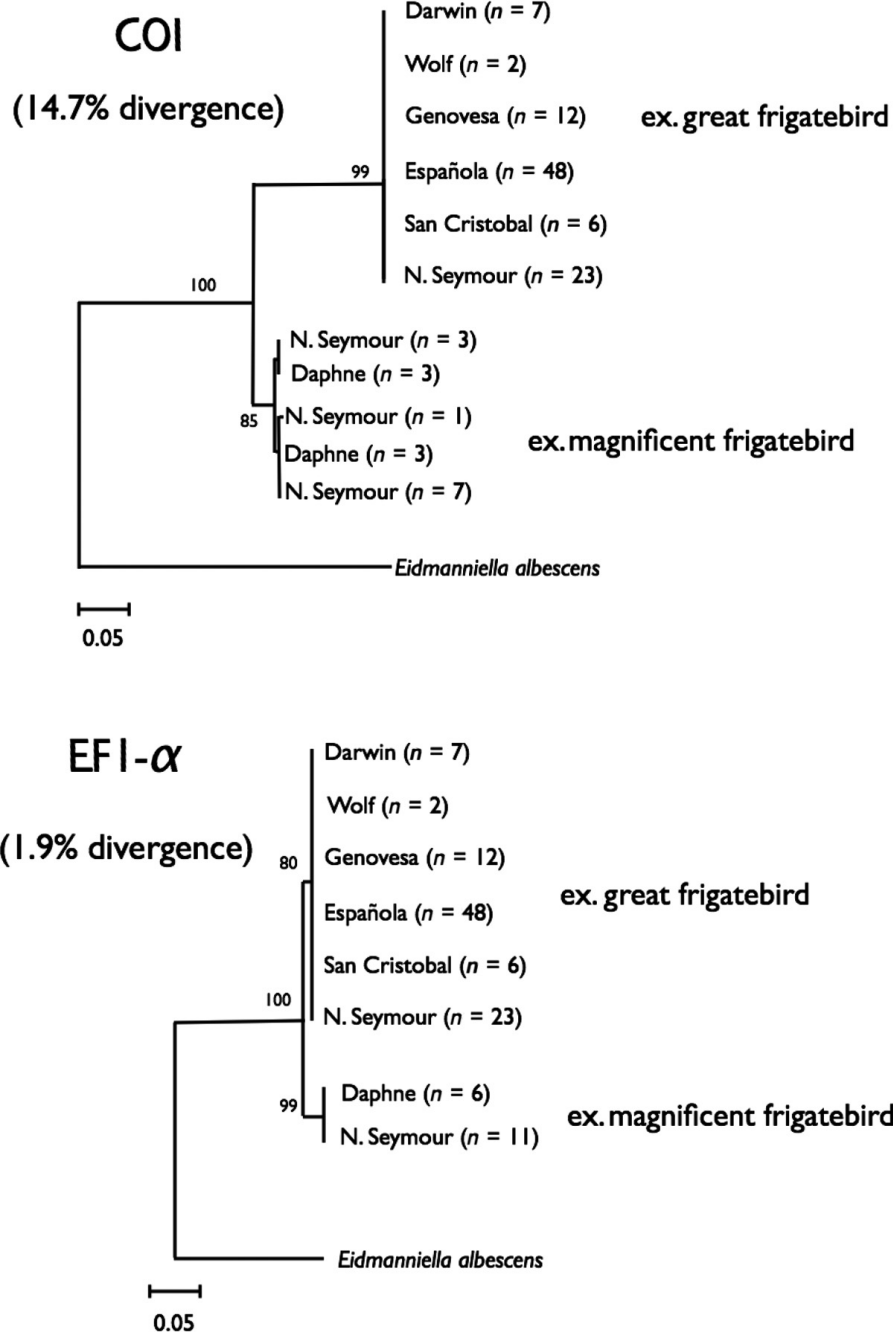
Maximum likelihood phylogenetic trees for the two genetic markers, *COI* and *EF1-α,* for *Fregatiella aurifasciata*. The number of parasites analyzed from each population is noted in parenthesis next to the island name.

We sequenced 115 individuals of *Fregatiella aurifasciata*, finding clear evidence of lineage sorting by host species (Fig. 3). The observed lineages from great frigatebird and magnificent frigatebird are 14.7% divergent (or 44-bp in a 300-bp fragment). The magnificent frigatebird lineage showed a mean genetic variation of 0.5%, whereas the great frigatebird lineage showed no genetic variation.

#### EF1-*α*

Sequences of *EF1-α* also clustered lice according to host species (Fig. 3). There was 1.9% genetic distance between these two groups (or 5-bp in a 270-bp fragment sequenced). The marker was monomorphic within each lineage.

Consistently, we found no evidence of lice from one lineage on the alternate host in both species of multihost parasites. Moreover, we did not find evidence of clustering based on island where the host was sampled, nor intraspecies clustering by geography within any of the host-specific lineages.

## Discussion

Two species of seabird lice from the Galapagos showed evidence of cryptic speciation and lineage sorting, even in cases where the potential for host switching and gene flow is high. In *Fregatiella aurifasciata,* two very distinct and genetically divergent monophyletic lineages were recovered from two different host species. Studies carried out on the *Pectinopygus* genus of ischnoceran ectoparasitic lice infecting frigatebirds are consistent with our results; ectoparasite diversification appeared to follow a pattern of cospeciation with the host species (Hughes et al. 2007). Results in these cases suggest that dispersal of lice between host species is limited enough such that distinct parasite lineages can emerge.

Similar to *F. aurifasciata*, *E. albescens* individuals showed clear lineage sorting when the mitochondrial marker was analyzed, but such differentiation was not as evident in the nuclear markers, which showed very little divergence overall. This lack of divergence of the nuclear genes is likely due to the much slower evolutionary rate of these nuclear genes in lice and insects in general as compared to mitochondrial genes (Johnson et al. 2003a,b; Lin and Danforth 2004). Studies of deeper evolutionary relationships of the *Eidmanniella* clade that include samples from hosts elsewhere and that directly test the timing of divergence will clarify this pattern and have the potential to distinguish between host switching and other scenarios. We recommend a taxonomic revision of both species, considering the degree of differentiation, which is in the range of what has been observed between different species in other lice genera (10–20%; Johnson et al. 2002).

Our results highlight the importance of genetic studies to understand and describe cryptic biodiversity of parasites (Poulin and Keeney 2008). Furthermore, detailed studies on the evolutionary history of these populations or species may lead to a better understanding of local adaptation and population dynamics and can provide relevant information to define management units to conserve not only taxonomic biodiversity but also unique evolutionary histories (phylogenetic diversity) as well (Waples and Gaggiotti 2006; Paisbøll et al. 2007).

In both parasite species, geography and immediate host community assemblage within a colony were irrelevant to patterns of genetic structure. We initially predicted that geography would be a significant factor in genetic clustering of parasites. Whiteman et al. (2007) showed how comparable ectoparasitic lice showed a stronger pattern of differentiation than their fragmented host population, using microsatellite markers. In our case, we were expecting that *E. albescens* found on the Nazca boobies would show some degree of genetic structure across islands similar to what is found in its host (Levin and Parker 2012). However, the resolution of the markers we used may have prevented detection of such differentiation (Gómez-Díaz et al. 2007).

The lineages of *E. albescens* from the blue-footed boobies and *F. aurifasciata* from great frigatebirds showed no clustering based on geography, which is consistent with the evidence from host population genetics (Taylor et al. 2011; Levin and Parker 2012).

We did not find any cases where an individual from one genetic lineage was found on the alternate host either in *F. aurifasciata* or in *E. albescens*. McCoy et al. (2005) found that local composition of a colony had no effect on the genetic differentiation of analyzed tick parasite, which is consistent with our findings. We did not find host switching even on islands where host colonies have some overlap such as Española in the case of the boobies, and North Seymour in the case of the frigatebirds. A caveat is that our sampling effort is a snapshot in a highly dynamic system, where seabird colonies in the archipelago are reported to change in species composition significantly across years (C. Valle, pers. comm.). Furthermore, we found that at fine scale, even on islands where the hosts are sympatric, there is low spatial overlap of nests across species. Both lice are relatively uncommon, with relatively low intensities of infection (Rivera-Parra et al. 2014). Thus, this fine-scale spatial separation together with the small number of individual lice that can “jump” from one host to the other may explain the absence of each lineage on the alternate host. Low louse population numbers and higher contact within host species than between host species could explain the pattern of lineage sorting by host observed in both lice species and the low intralineage genetic diversity. Moreover, the whole life cycle of these parasites is about 3 weeks (Price et al. 2003); thus, low potential for gene flow and short generation times may further reinforce the isolation pattern detected in this study. In addition, amblyceran lice have the potential to interact with the host's immune system (Whiteman et al. 2006), and thus, there may be local adaptation by these lice to host defenses. Selection combined with dispersal limitation might be reinforcing these patterns.

Rivera-Parra et al. (2014) and Palma and Peck (2013) did not find *E. albescens* on red-footed boobies from the Galapagos archipelago, even though this seabird species is a documented host for *E. albescens* elsewhere (Price et al. 2003). Our results suggest a previously undescribed lineage specificity among these lice. We encourage further analysis of *E. albescens,* including parasites from the red-footed boobies from locations outside the Galapagos, to examine whether there is a specific lineage for this host species elsewhere that was lost during the colonization process.

## Conclusion

Our study shows how detailed genetic studies on multihost parasite species can greatly increase our understanding of biodiversity and speciation even when morphological differences are not evident. Furthermore, parasite diversity seems to depend primarily on host diversity rather than on geography or the local host community composition. Our study suggests that this intimate host–parasite relationship prevents gene flow across parasites found on different host species, promoting the divergence of host-specific lineages. This study shows snapshots of this process, with one parasite showing marked genetic divergence (*Fregatiella aurifasciata*) in both mitochondrial and nuclear markers, and another in the early steps of differentiation showing strong evidence of lineage sorting in the presumably faster evolving mitochondrial marker and only in one of two more slowly evolving nuclear gene regions (*Eidmanniella albescens*).
